# Elucidation of the structural stability and dynamics of heterogeneous intermediate ensembles in unfolding pathway of the N-terminal domain of TDP-43†

**DOI:** 10.1039/c8ra03368d

**Published:** 2018-05-30

**Authors:** Amresh Prakash, Vijay Kumar, Naveen Kumar Meena, Andrew M. Lynn

**Affiliations:** School of Computational & Integrative Sciences, Jawaharlal Nehru University New Delhi-110067 India amreshprakash@jnu.ac.in; Centre for Interdisciplinary Research in Basic Sciences, Jamia Millia Islamia Jamia Nagar New Delhi-110025 India vijay9595@st.jmi.ac.in

## Abstract

The N-terminal domain of the RNA binding protein TDP-43 (NTD) is essential to both physiology and proteinopathy; however, elucidation of its folding/unfolding still remains a major quest. In this study, we have investigated the biophysical behavior of intermediate ensembles employing all-atom molecular dynamics simulations in 8 M urea accelerated with high temperatures to achieve unfolded states in a confined computation time. The cumulative results of the 2.75 μs simulations show that unfolding of the NTD at 350 K evolves through different stable and meta-stable intermediate states. The free-energy landscape reveals two meta-stable intermediates (I_N_ and I_U_) stabilized by non-native interactions, which are largely hydrophilic and highly energetically frustrated. A single buried tryptophan residue, W80, undergoes solvent exposure to different extents during unfolding; this suggests a structurally heterogeneous population of intermediate ensembles. Furthermore, the structure properties of the I_N_ state show a resemblance to the molten globule (MG) state with most of the secondary structures intact. The unfolding of the NTD is initiated by the loss of β-strands, and the unfolded (U) states exhibit a population of non-native α-helices. These non-native unfolded intermediate ensembles may mediate protein oligomerization, leading to the formation of pathological, irreversible aggregates, characteristics of disease pathogenesis.

## Abstract 2

The N-terminal domain of the RNA binding protein TDP-43 (NTD) is essential to both physiology and proteinopathy; however, elucidation of its folding/unfolding still remains a major quest.

## Graphical abstract

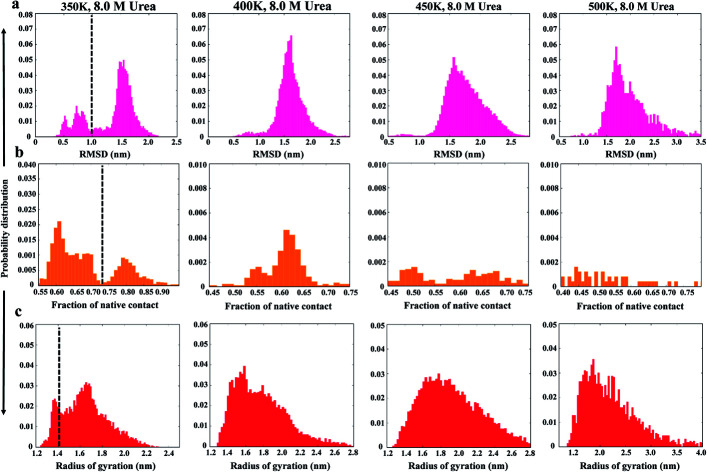

## Introduction

Amyotrophic lateral sclerosis (ALS) and frontotemporal dementia (FTD) are two closely related debilitating adult-onset neurodegenerative diseases.^1–3^ The mislocalization and cytoplasmic ubiquitinated inclusions of TAR DNA binding protein-43 (TDP-43) are linked to majority of ALS and FTD.^4^ Several studies indicate that the possible mechanism of TDP-43-induced neurotoxicity includes a complex interplay between the loss of cellular functions and gain of toxic functions of TDP-43.^5–7^ TDP-43 belongs to a highly conserved heterogeneous nuclear ribonucleoprotein (hnRNP) that binds TG/UG repeat nucleic acids and plays an important role in RNA metabolism.^8,9^ TDP-43 comprises four functional domains: an N-terminal domain (NTD), two RNA recognition motifs (RRM1 and RRM2), and a glycine-rich, disordered C-terminal domain (CTD). The CTD is a low complexity, prion-like domain that harbors most of the ALS-linked mutations.^10^ This domain mediates protein–protein interactions and also self-interacts and undergoes phase separation and aggregation.^11–13^

After being ignored for almost six years since the discovery of TDP-43, the NTD has drawn significant attention recently; in the past few years, many studies have revealed that the NTD plays a role in the physiological functions of TDP-43 (ref. 14–18) as well as in its pathological aggregation and neurotoxicity.^17,19–21^ The solution and crystal structures of the NTD have been elucidated by NMR and X-ray crystallography. In 2014,^14^ Qin *et al.* reported that the NTD (residues 1–102) remains in both well-folded and unfolded structures in equilibrium, and the folded structure adopts an ubiquitin-like fold that binds single-stranded DNA. The first solution-structure of the stably folded NTD (residue 1–77) was solved by Mompean *et al.* in 2016.^22^ The structure of the NTD is a canonical β-barrel structure consisting of six β-strands and a short α-helix with a topology of β1β2 αβ3β6β4β5 (PDB ID: 2N4P). The same research group later published the solution structure of a longer NTD (residues 1–102; PDB ID: 5MRG) and described the atomistic model structure for a dimeric NTD observed *in vitro*.^23^ Very recently, Afroz *et al.* solved the crystal structure of the NTD for the first time and reported that NTD-mediated TDP-43 oligomerization results in the physiological oligomers of TDP-43.^24^

The NTD domain is highly conserved among eukaryotes^25^ and is stable and well-folded at pH 2.0 to 8.6 and temperatures of 5 °C to 40 °C, including physiological conditions.^22,23,26^ Many recent structural and functional studies of the NTD were performed on His-tagged constructs.^14,18,22–24^ These studies clearly showed that the NTD is stably folded and functional. Recently, Tsoi *et al.*^26^ also showed that NTD_1–80_ without His-tags adopts a similar fold to Mompean's His-tagged NTD_1–77_.^22^

Mompean *et al.*^22^ reported that the conformational stability of the NTD is highest at acidic pH and decreases with increasing pH. However, the protein becomes unfolded only at pH > 10.0 because of mutual repulsion of anionic residues (∼12 in number). Recently, Tsoi *et al.*^26^ showed that at low pH, the NTD becomes destabilized, which leads to its monomeric form. They reported the presence of multiple native thermodynamic states (*i.e.* N1 and N2) at physiological conditions and suggested that these two native states correspond to pH-dependent protonation–deprotonation of the single His 62. Several studies have reported an interesting dichotomy in TDP-43, where the NTD regulates normal TDP-43 structure and function^16,24,27^ while also participating in driving TDP-43 aggregation in TDP-43 proteinopathies.^15–17,19^ TDP-43 forms physiological and reversible oligomers mediated by the NTD, and these physiological oligomers may serve as precursors for pathological and irreversible protein inclusions. Thus, the physiological oligomers of TDP-43 form when the NTD is stable and well folded. However, unfolding of the NTD results in the formation of pathological inclusions.^14,28^ In this regard, biophysical behavior studies of folding dynamics and structural stability analysis of the NTD will be beneficial to understand the biological roles played by TDP-43 under physiological and pathological conditions.

High-temperature unfolding simulations are a useful system to study protein unfolding on computationally accessible time-scales.^29^ By starting from a relevant conformational state, these high-temperature MD simulations allow easier access to relevant intermediate states and can demonstrate that both native and non-native interactions contribute to stability. A large number of high-temperature MD experiments have demonstrated that the pathways of protein folding and unfolding significantly obey the principle of microscopic reversibility.^30–32^ Also, the unfolding pathways for the same protein do not depend on the force-fields utilized in MD studies.^33^ Indeed, the unfolding pathway has been demonstrated to be temperature-independent.^30,34^ Therefore, high-temperature MD simulations have been employed to investigate the unfolding pathways or stabilities of several proteins.^35–39^

To increase the rate of the unfolding process, both experimental and computational methods utilize high temperature, high pressure, low pH, or chemical denaturants (guanidinium chloride or urea). In the present work, we have employed all-atom MD simulations to obtain detailed insights into the unfolding and thermostability of the NTD in 8 M urea at 300 K, 350 K, 400 K, 450 K and 500 K. The results of the cumulative microsecond simulations indicate (i) the presence of stable intermediates (I_N_ and I_U_) and the transition state (TS) along the unfolding pathway; (ii) the formation of a molten globule (MG) during an early unfolding intermediate, I_N_; (iii) conformationally heterogeneous I_U_ stabilized by non-native contacts and hydrogen bonds; (iv) initiation of unfolding through disruption of β3; and (v) the presence of residual structures in the unfolded state. This study may provide further insights into the unfolding of the N-terminal domain of TDP-43, which contributes to protein aggregation and is thus implicated in pathological roles in neurodegenerative diseases.

## Materials and methods

### MD simulations

All-atom MD simulations were performed using Gromacs v5.1.4 with the CHARMM27 force field and SPC216 as a water model. The initial co-ordinates of the NTD of TDP-43 (PDB ID: 2N4P) were obtained from the Protein Data Bank.^22^ As a control simulation, the protein was solvated in pure water in a cubic box with a volume of 6 nm^3^. To conduct simulations in aqueous urea, the protein was placed centrally in a pre-equilibrated box (volume 6 nm^3^) of 8 M urea (865 molecules of urea and 4521 molecules of water). The parameters and topology files to prepare the aqueous mixture of urea molecules were taken from Camilloni *et al.*^35^ This parameter has been extensively used by our group as well as others to study protein unfolding.^40–44^ To neutralize the system, seven molecules of Na+ ions were added to the bulk (solvent). The prepared systems were minimized using the steepest descent method followed by the conjugant gradient method, with 10 000 steps for both. The system was equilibrated in two steps, NVT and NPT; each step was performed for 500 ps. Periodic boundary conditions (PBC) were exerted in all three *x*, *y* and *z* directions. Berendsen thermostat and Parrinello–Rahman pressure coupling were used to maintain the temperature and pressure, respectively. The bond lengths and hydrogen atoms were constrained using the LINC algorithm^45^ and particle mesh Ewald method;^46^ a grid space of 1.0 nm was used to evaluate the electrostatic interactions, and the van der Waals interactions were evaluated using the LJ potential with cutoff 1.2 nm. Finally, the NPT ensemble was used for the production run for 500 ns in pure water and 8 M urea at 300 K, with an integration time step of 2 fs; the coordinates and trajectory for velocity and energy were recorded for every 10 ps, respectively. To accelerate the unfolding kinetics, four independent runs were also carried out at 350 K, 400 K, 450 K and 500 K in 8 M urea. The analyses on the obtained trajectories were performed using Gromacs utilities and MDTraj-based Python scripts.^47^ The tools provided by the GROMACS program package were utilized to analyze different MD trajectories. The PyMOL (DeLano Scientific) and XMGrace programs were used to analyze and prepare publication-quality figures.

### Fraction of native contacts

A protein in its folded native state is stabilized energetically and is driven by various contacts that are partially or fully but collectively involved in interactions to maintain the appropriate fold. We measured the fraction of native contacts as previously described.^48^ The native pairs were defined as all the pairs of heavy atoms *i* and *j* belonging to residues *θ*_*i*_ and *θ*_*j*_ that are in contact, provided that |*θ*_*i*_ − *θ*_*j*_| > 3 and the distance between *i* and *j* is less than 4.5. Also, the fraction of native contacts is defined as:1
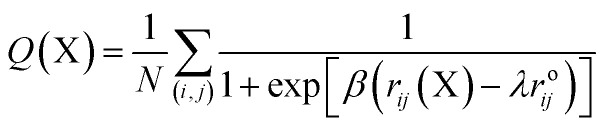
where *N* is the pairs of native contacts (*i*,*j*), *r*_*ij*_ (X) is the distance between *i* and *j* in configuration X, *r*^o^_*ij*_ is the distance between *i* and *j* in the native state, *β* is the smoothing parameter with a value of 5 Å, and the factor *λ* accounts for fluctuations when contact is formed; it is taken as 1.8.

### Free energy contour maps of the NTD

The free energy contour maps were established by calculating the normalized probability distribution based on a set of order parameters. We defined the free energy based on probability distribution (*P*) using the relation *F* = −*RT* ln *P*.

Here, we described the free energy map as a function of two order parameters, that is, the root mean square deviation (RMSD) and fraction of native contacts (*N*_c_). We also constructed an additional free energy contour map as a function of the radius of gyration (*R*_g_) and *N*_c_ to validate the analysis.

### Residual frustration analysis

The Woylness group developed an algorithm to determine local frustration in a protein residue which indicates whether a contact between residues is energetically optimized in the folded state.^49^ This algorithm is now implemented in a web server, “Frustratometer”;^50,51^ it examines pairwise interactions between residues and evaluates the total energy changes. In this work, we used the “configurational frustration index”, in which the decoy set involves randomizing the identities as well as the distance and the density of interacting amino acids *i* and *j*. This scheme effectively determines the native pair with respect to a set of structural decoys that may be observed during the folding events. Accordingly, energetically favorable contacts between native residue pairs are considered to be minimally frustrated, whereas energetically unfavorable contacts in the native state are considered to be highly frustrated.

We analyzed the frustration in NTD during unfolding, and the minimally and highly frustrated contacts are depicted on the lowest-energy structure from the Boltzmann-reweighted structures from the MD simulations.

## Results and discussion

Multiple unconstrained MD simulations of NTD solvated in aqueous 8 M urea solution at different temperatures (300 K, 350 K, 400 K, 450 K, and 500 K) were performed and analyzed by the simultaneous use of several parameters to enable a detailed characterization of the unfolding process of NTD. The simulations were carried out for 500 ns in water (at 300 K) and 8 M urea (at 300 K to 450 K) and for 250 ns (at 500 K); thus, the total simulation time was 2.75 μs.

Control MD simulations were carried out in water and 8 M urea at 300 K for 500 ns, respectively (Fig. S1†). The results clearly showed that the NTD in both water and 8 M urea at room temperature was stable and close to its native structure without any unfolding propensity. In water, the Cα root mean-square-deviation (RMSD) did not deviate greatly and stabilized around a value of <1.0 nm; meanwhile, the radius of gyration (*R*_g_), fraction of native contacts (*N*_c_) and solvent accessible surface area (SASA) of the NTD also remained stable during the simulations. However, in 8 M urea, slight increases in RMSD, *R*_g_ and SASA were observed along with a slight decrease in *N*_c_. Thus, to accelerate the unfolding kinetics, the simulations in the presence of 8 M urea were augmented with higher temperatures;^30,39,52^ the details of the conformational changes are summarized in ESI Table 1.†

### Temperature dependence of conformational dynamics

**Fig. 1:**
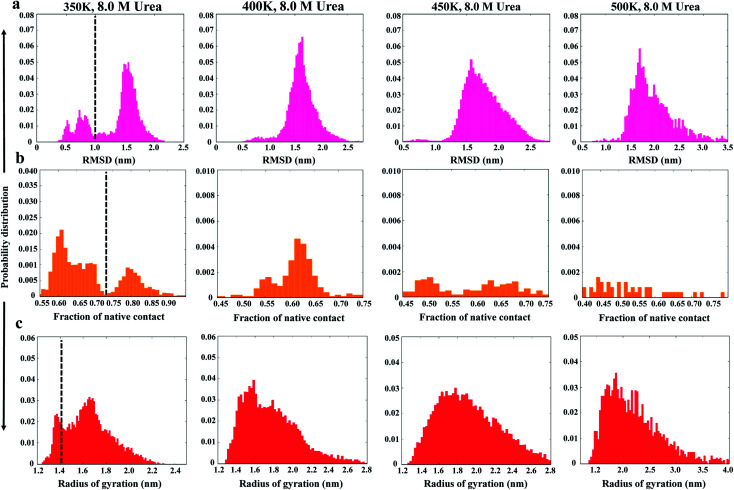
Probability distributions of structural parameters: (a) Cα-RMSD, (b) number of native contacts (*N*_c_), and (c) radius of gyration (*R*_g_) in 8 M urea at 350 K (left panel) to 500 K (right panel). The dashed line defines the two different conformational states as native and non-native states.

As a first step in characterizing the unfolding dynamics of the NTD, we studied the temperature dependence of several structural parameters obtained from MD trajectories in the range of 300 to 500 K. Fig. 1 summarizes the temperature dependence of the Cα RMSD (Fig. 1a), *N*_c_ (Fig. 1b), and *R*_g_ (Fig. 1c). We also show the convergence of these parameters in the ESI, Fig. S2.†

As can be seen from the results, the NTD structure deviates greatly with increasing temperature, indicating progressive unfolding of the NTD. The shift in the probability distribution of RMSD toward higher values (Fig. 1a) and the shift toward lower values in *N*_c_ (Fig. 1b) clearly indicate loss of the native structure. At 350 K, the RMSD and *N*_c_ distributions reveal a distinct bimodal nature; this indicates the presence of two major states, native and non-native. The distribution at higher temperatures gradually shifts to a non-native state. Interestingly, the distributions also demonstrate the appearance of an intermediate state around an RMSD of ∼0.8 to 1.0 nm and an *N*_c_ of ∼50, which is largely populated at 350 K; this suggests that the transition occurs at this temperature. Thus, at 350 K, *N*_c_ displays higher fluctuations during the simulation, indicating the presence of several intermediate states between the folded and unfolded states (ESI, Fig. S2b†).

Alternatively, *R*_g_ remains relatively unchanged or slightly decreases with increasing temperature (Fig. 1c and S2c†), indicating that the protein assumes a compact molten globule structure upon the initial melting of the native structure. The transition from a compact molten globule-like state to a fully unfolded state (with a large *R*_g_) occurs at much higher temperatures (450 to 500 K).

### Unfolding of NTD monitored by solvent accessibility of Trp80

**Fig. 2:**
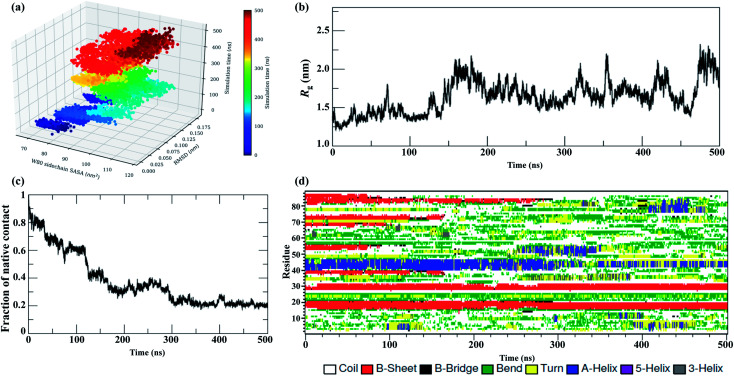
Unfolding pathways of the NTD in 8 M urea at 350 K. (a) Evolution of the solvent-exposed surface area, SASA, of the W80 side chain and Cα-RMSD during unfolding. Each point on this plot is colored according to its time of occurrence according to the color scale shown. Time evolution of (b) *R*_g_, (c) *N*_c_, and (d) secondary structures.

The MD trajectories in 8 M urea with different temperatures were then used to determine the solvent accessibility of an unfolding probe, Trp80 residue (Trp68 in Uniprot id: Q13148). The residue Trp80 (W80) is buried within the hydrophobic core of the protein as determined by NMR spectroscopy.^22^ The simulations at 350 K were used to investigate the urea-induced unfolding of the NTD in detail, with emphasis on characterization of the molten globule-like intermediate state. The results from the MD run at 350 K are shown in Fig. 2. The results at all other temperatures (300, 400, 450, and 500 K) in 8 M urea are shown in the ESI, Fig. S3–S6.†

Fig. 2a shows the evolution of the side chain SASA of W80 along with the Cα RMSD during a 500 ns trajectory at 350 K. In the native state, W80 is solvent-exposed, with a side chain SASA of ∼70 nm^2^. At 350 K, W80 undergoes different extents of solvent exposure at different stages of unfolding, consistent with an experimentally observed decrease in fluorescence.^22,26^ The first transition of solvent exposure (SASA ∼ 80 to 95 nm^2^) is evident for a short time scale of 50 ns (from ∼80 to 130 ns) in a native-like ensemble with RMSD < 0.12 nm. This ensemble is referred to as I_N_ hereafter. The side chain SASA of W80 increases as I_N_ unfolds further on a longer time scale of 100 ns (∼200 to 300 ns). This partially unfolded intermediate ensemble (referred to as I_U_) is populated with an RMSD of 0.15 nm. The I_U_ ensemble further unfolds to the unfolded state, U, with RMSD > 0.17 nm. This unfolded state displays the transition of W80 between partially buried and solvent-exposed states. The fluctuation of SASA in this state is larger than both I_N_ and I_U_, suggesting that the environment around W80 is more malleable in the U state. Previous fluorescence experiments^22^ showed that solvent exposure of W80 is accompanied by a relatively small red shift in the *λ*_max_ of W80 (from 328 to 350 nm); however, the reason for this shift is not clear. Here, we show that W80 in the U ensemble is fully solvent exposed in only a fraction of U molecules explored during the last ∼100 ns of the simulation (400 to 500 ns).

I_U_ is a more structurally loose ensemble than I_N_ and displays a loss of most of the *N*_c_ and secondary structures. Moreover, I_U_ is much longer-lived than I_N_ (in all temperature simulations), suggesting that most of the decrease in fluorescence intensity observed throughout the experiment is contributed by the I_U_ ensemble. Further, W80 was also found to be transiently and infrequently buried in the I_U_ and U ensembles, suggesting that the conformational ensembles are structurally heterogeneous and are populated at different stages of unfolding.

In addition to W80, a tyrosine residue (Y55) also shows transient fluctuations between partially buried and fully solvent-exposed states (ESI, Fig. S7†), suggesting structural heterogeneity within the I_U_ ensemble.

The evolution of *R*_g_ (Fig. 2b) further suggests that the I_N_ ensemble in which W80 is exposed is highly compact in nature, with *R*_g_ ∼ 1.30 nm, and remains native-like; this is also suggested by the RMSD (<1.0 nm). In contrast, the I_U_ ensemble shifts slightly towards the unfolded side, with *R*_g_ > 1.70 nm and RMSD > 1.50 nm. We observed transient drops in the *R*_g_ and RMSD values, which suggests non-specific collapse of the structure. The evolution of *N*_c_ (Fig. 2c) further confirms that I_N_ has a native-like structure (*N*_c_ ∼ 0.6), whereas the I_U_ ensemble is more unfolded (*N*_c_ ∼ 0.35).

Interestingly, a comparison of the evolutions of the W80 side chain SASA and RMSD in higher temperature simulations (ESI, Fig. S3–S6†) displays a similar manner of unfolding, *i.e.* a compact molten globule native-like I_N_ structure early in the unfolding process followed by a long-lived partially unfolded I_U_ conformation and, eventually, the fully unfolded U conformation.

The results of the simulations presented in Fig. 2 and S3–S6† show that solvent exposure of W80 can occur on different time scales and is dependent on the temperature, suggesting that the reaction is non-cooperative and is not all-or-none. However, it remains to be determined whether this non-cooperativity is due to the presence of urea or increased temperature.

The evolution of the secondary structure was calculated using the DSSP program to obtain a detailed understanding of the structural changes during the unfolding process. Fig. 2d shows that the secondary structure of the NTD at 350 K remains fairly stable up to ∼100 ns, although the number of native contacts decreases to 60%. The unfolding process initiates with the disruption of β3 and β4 along with the loss of turns and bridges between these two strands. As unfolding continues, β6 is lost completely, followed by massive loss of the α-helix at the midpoint of the simulation. However, the N-terminal strands β1 and β2 remain mostly intact during the simulation.

The early unfolding steps at higher temperatures (450 K to 500 K) involve the sequential loss of β strands at the C-terminal, followed by loss of N-terminal strands and the α-helix (Fig. S3–S6†). Formation of the non-native helix was also evident during the simulation. Thus, the β-sheets were lost more quickly than the α-helix in urea at higher temperatures. These results are in good agreement with previously reported data.^35,44^

### Tertiary contact map

**Fig. 3:**
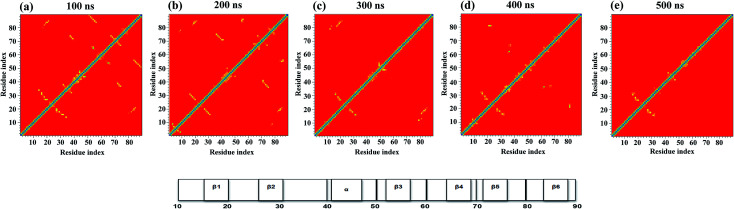
Tertiary contact map of the NTD unfolding pathway in 8 M urea at 350 K as seen at different time points. (a) 50 ns represents the N state, (b) 100 ns represents the I_N_ state, (c) 200 ns represents the I_U_ state, (d) 400 ns represents the transition state, and (e) 500 ns represents the U state. The horizontal bar on the right denotes the secondary structures present in the original NMR structure.

Fig. 3 shows the pairwise contact maps for the N → I_N_ → I_U_ → U unfolding process observed at 350 K. This enables us to obtain insights into the details of initial unfolding events and highlights the differences in the contacts formed during different stages of unfolding. A contact map of Cα atoms (≤6.0 Å) was used to characterize the unfolding of the NTD by showing the formation and loss of contacts between the residues.

The contact map analysis implies that the loss of tertiary contacts during the unfolding pathway is gradual; however, the unfolding is initiated with weakening of the long-range contacts between β3–β6 and short range contacts between β4–β5 at 100 ns in the simulation (Fig. 3a). The contacts between β3 (52–56) and β-bridge residues (64–65) are also lost. Weakening of loop contacts (32–38) is observed in the I_N_ ensemble and becomes more prominent in I_U_. Moreover, gains of some non-native contacts between the α-helix and β-hairpin residues (*i.e.* 40–70; 41–71) between the loop and β5 (*i.e.* 38–72) and the β-bridge and β4 (*i.e.* 64–68) are observed in the I_N_ ensemble. However, long-range contacts between β1–β6 and between the α-helix and β5, as well as short-range contacts involving the N-terminal strand β1, β2 and the β-hairpin, remain intact (Fig. 3b); this indicates the preservation of this N-terminal core in the I_N_ state.

During the next stage of unfolding, complete loss of long-range contacts between strands β3–β6 and short-range contacts between β4–β5 is clearly observed in I_U_. Furthermore, the contacts involving the α-helix and β5 are also disrupted completely. The contacts at both termini of the α-helix are weakened. Loss of many non-native contacts present in the I_N_ ensemble is observed in I_U_, along with the formation of some new non-native contacts between the turn and β3 residues (*i.e.* 48–52 and 48–54). However, the long-range contacts between β1–β6, short-range contacts between β1–β2, and α-helical contacts remain intact (Fig. 3c), indicating that these N-terminal contacts are preserved in both the I_N_ and I_U_ ensembles.

The disruption of long range contacts between β1–β6 along with the appearance of new non-native contacts is observed in the transition state (∼300 to 400 ns) between the I_U_ and U states. Interestingly, many short-range non-native contacts appeared between residues of β3 (52), β4 (67–68), bridge (37–38), loop (32–34), turn (47–50), and β5 (72), indicating the formation of a non-native unfolded transition state ensemble (Fig. 3d).

The I_U_ ensemble further unfolds with predominant loss of the α-helix. However, the contacts involving β1, β2 and the β-hairpin remain intact, indicating the presence of a residual structure even in the unfolded conformation with *N*_c_ < 20% (Fig. 3e). The existence of a residual tertiary structure in the unfolded state may be due to the change in the environment of the aromatic residues (Trp, Tyr, and Phe) as the protein unfolding commences (see Fig. 2a and S7†). Also, a transient drop in *R*_g_ for the U ensemble is consistently observed, suggesting the presence of a collapsed structure in the U ensemble (Fig. 2b). The presence of residual structure in denatured states has been previously reported for many proteins.^52–55^

**Fig. 4:**
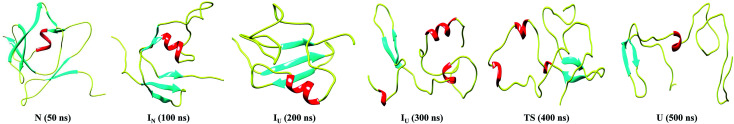
Snapshots corresponding to the N, I_N_, I_U_, TS and U conformational states observed during the unfolding pathway of the NTD at 350 K.

Furthermore, snapshots of the unfolding events at 350 K are shown in Fig. 4. The structural snapshot at 50 ns corresponds to a native (N) structure, where all the native tertiary contacts and secondary structures remain intact. Moving from the N state to I_N_ at 100 ns, we can clearly observe disruption of the β3 and β4 strands; however, the N-terminal strands β1 and β2 and the α-helix remain intact. The conformation representing the I_U_ ensemble (200 to 300 ns) is largely unfolded, with melting of the α-helix along with loss of β5 and β6. The structural snapshot of the transition structure (400 ns) shows further loss in the single α helix, along with the presence of non-native helical structures. The unfolded ensemble at 500 ns is characterized by the presence of largely unfolded and disordered structures. The unfolded state still contains the residual structure with intact β1 and β2, and the α helix is almost completely melted. Snapshots depicting the complete unfolding pathway of the NTD at a higher temperature (400 K) are shown in the ESI, Fig. S8.†

### Free-energy contour map of the NTD reveals stable intermediates

**Fig. 5:**
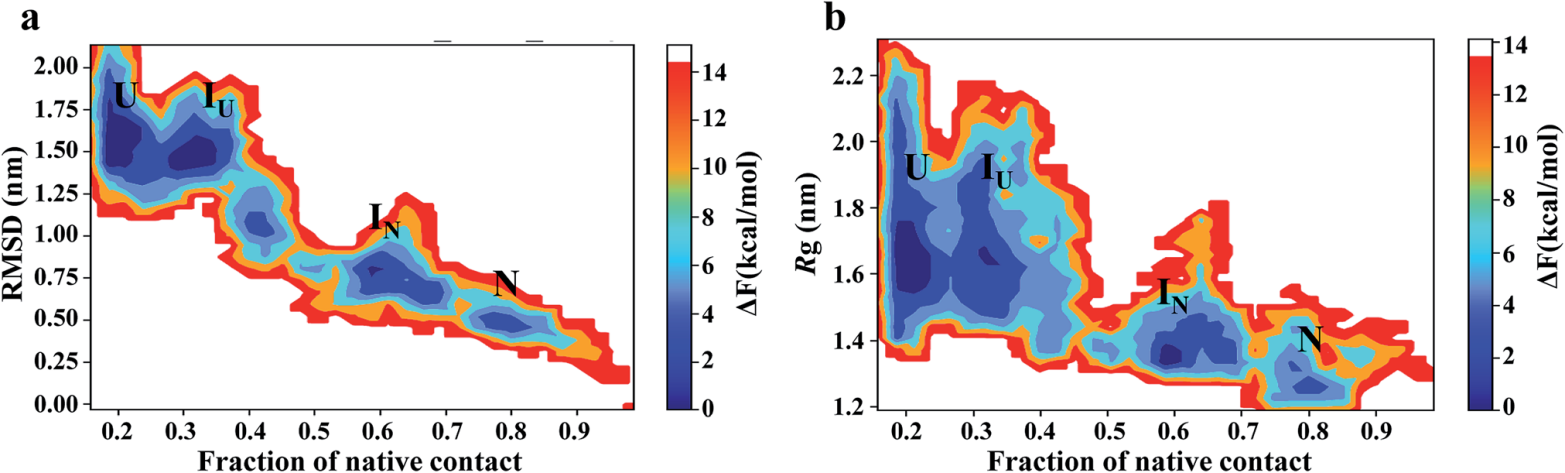
Free energy contour maps constructed from (a) Cα-RMSD *versus* native contact (left panel) and (b) *R*_g_*versus* native contact (right panel) in 8 M urea at 350 K. The color is scaled according to kcal mol^−1^. Key minima (basins) in the maps are marked.

To map the unfolding pathway in 8 M urea at temperatures from 350 to 500 K, a two-dimensional free energy landscape (FEL) contour map was constructed from the joint probability distribution (*P*) of *N*_c_ with RMSD and *R*_g_ using the equation *F* = −*RT* ln *P*. The FEL plot shows that the transition from the native folded state to the unfolded state follows a minimum energy pathway evolving through different intermediates (Fig. 5). At 350 K, the highly rugged FEL shows the presence of three distinct global minima in addition to two well-resolved local minima, indicating the presence of several intermediates between the folded and unfolded conformations (Fig. 5a). These different energy minima are well separated by a relatively small energy barrier of ∼7.0 kJ mol^−1^. The plot shows that the protein quickly moves out from the native basin and explores a broad basin with an RMSD of ∼0.75 nm and *N*_c_ of ∼0.6 during 130 ns of the simulation; this corresponds to the I_N_ ensemble. The I_N_ ensemble is native-like, with similar *R*_g_ and *N*_c_ values (Fig. 5b).

The protein then visits a much broader energy basin *via* a transition state; within this broader phase region, the protein explores two distinguishable minima separated by a very small energy barrier of <4.0 kJ mol^−1^. The first minimum (referred to as I_U_) is achieved from 200 to 300 ns of the simulation, with an RMSD of ∼1.50 nm and *N*_c_ of ∼0.35. I_U_ is slightly swollen compared to the N state, with *R*_g_ ∼ 1.7. The second minimum (referred to as U) displays a largely populated phase space with an RMSD of ∼1.50 to 1.75 nm and the presence of ∼20% of native contacts.

Thus, the I_N_ ensemble is a short-lived intermediate ensemble that is observed for 50 ns (80 to 130 ns) and is structurally compact (*R*_g_ ∼ 1.3 nm) and native-like (*N*_c_ ∼ 0.6) with most of the secondary structures intact but with a significant loss of tertiary contacts. These structural features are characteristic of the molten globule state. However, the I_U_ ensemble is a looser structure (*N*_c_ ∼ 0.35; *R*_g_ ∼ 1.8 nm), with significant loss of secondary and tertiary structures. FEL at higher temperatures (400 K to 500 K) exhibited broad prominent minima with RMSD > 1.5 nm and *N*_c_ ∼ 0.1, representing the unfolded (U) state of the protein (Fig. S9†). For simplicity, we have shown two FEL contour maps in each row. The first column depicts the FEL calculated as a function of the *N*_c_ and RMSD pair, whereas the second column shows the FEL against *N*_c_ and *R*_g_. The overlap between these two plots clearly suggests excellent agreement for the reported configurations.

**Fig. 6:**
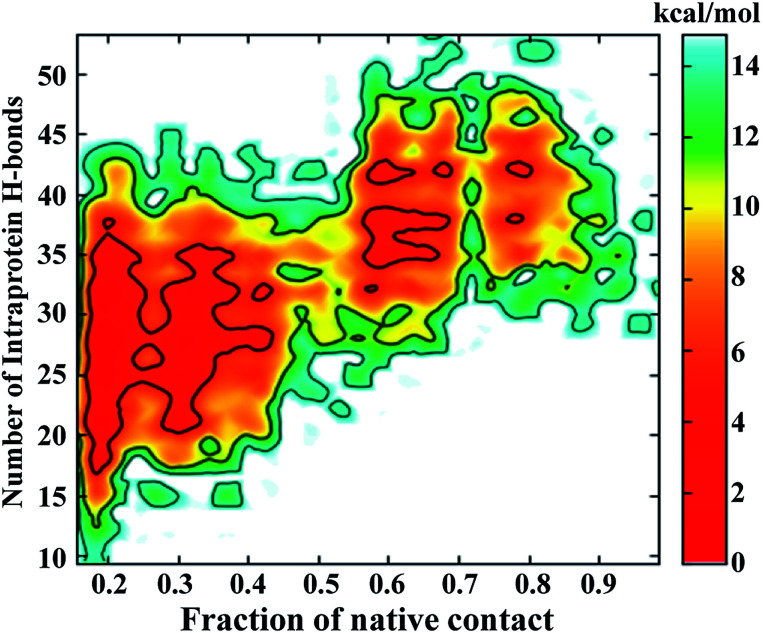
FEL of the number of intraprotein hydrogen bonds *versus* the number of native contacts at 350 K.

The FEL involving the number of intraprotein hydrogen bonds and the number of native contacts at 350 K is shown in Fig. 6. Remarkably, the number of intraprotein hydrogen bonds decreases from ∼40 to ∼20 along the unfolding pathway as the number of native contacts decreases from ∼70% to ∼20%. Thus, the significant decrease in non-native hydrogen bonds suggests the absence of misfolded states during the unfolding of NTD. However, at higher temperatures (400 K to 500 K), intraprotein hydrogen bonds are retained as the protein becomes completely unfolded, indicating the persistence of non-native hydrogen bonds (Figure S10†).

### Disruption of the hydrophobic core leads to solvent exposure of W80 during unfolding

**Fig. 7:**
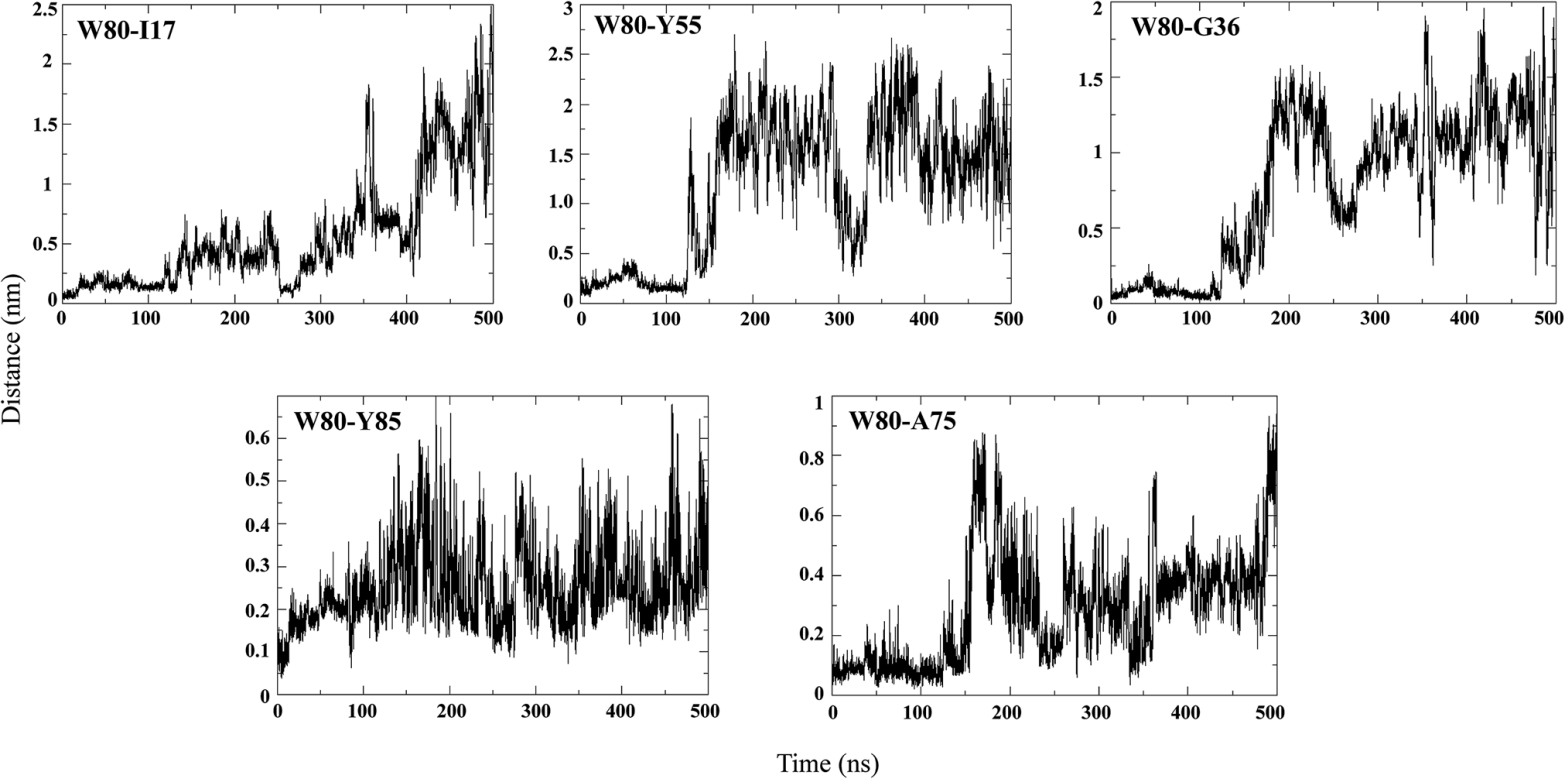
Pairwise distance distribution of representative hydrophobic contacts during the simulation at 350 K.

An NMR study of the NTD (PDB ID: 2N4P)^22^ showed that W80 lies in the centre of the hydrophobic core of the protein and is involved in contacts with Ile17, Gly36, Tyr55, Ala75 and Tyr85. Hence, the pairwise distance distributions between these contacts were monitored during different stages of unfolding (Fig. 7). In the N state (0 to 50 ns), all the reported W80 contacts are within a distance of 0.5 nm, indicating the stability of the structure. In the I_N_ ensemble (50–130 ns), these contacts were found to be stable up to ∼110 ns, after which the inter-atomic distances between them increased slightly; the maximum change in pair-distance was observed between W80–Y55. These results thus signify that the I_N_ ensemble is native-like. The unfolding of I_N_ to I_U_ (200 to 300 ns) is accompanied by disruption of many of these contacts, with the maximum loss observed for W80–Y55. The contacts between W80–I17, W80–Y85 and W80–A75 are maintained. As unfolding progresses, the long-range contacts between W80–I17, W80–Y55 and W80–G36 are greatly disrupted. The short-range contacts between W80–Y85 and W80–A75 remain somewhat stable. As mentioned above, W80 explores both partially buried and solvent-exposed states in the I_N_ and U ensembles, which is evident from the pairwise distance distribution among W80 contacts.

### Structural basis of the higher stability of the NTD

The intermediate state, I_U_, is moderately stabilized by the preservation of the intraprotein hydrogen bonds (due to the presence of intact β1 and β2 during unfolding) and the intramolecular contacts as well as the presence of partially buried non-polar residues W80 and Y55 in the side chain. To analyze the physico-chemical nature of the interacting residues, the COCOMAPS server^56^ was used. The inter-atomic contact maps of the NTD unfolding ensembles are shown in the ESI, Fig. S11,† where the residues are said to be in contact when at least two heavy atoms are separated by ≤5 Å.

As can be seen from the results, the contact map of N is essentially hydrophobic in nature, whereas the contacts in I_U_ are predominantly hydrophilic in nature. The number of hydrophilic contacts in the I_U_ ensemble is significantly higher than in the N and I_N_ states, and the hydrophobic interactions are larger compared to the I_N_ ensemble. In contrast, the contacts in I_N_ exhibit equal numbers of hydrophilic and hydrophobic interactions. This result suggests that I_U_ is stabilized by the presence of greater non-native hydrophilic–hydrophilic and hydrophobic–hydrophobic interactions. We therefore suggest that the stability of the intermediates causes NTD to be highly stable both thermodynamically and kinetically.

### Distribution of frustration in the protein

The concept of frustration was originally introduced by Wolynes *et al.* to describe the protein folding landscape.^49^ According to the principle of minimal frustration, the protein folding process is considered to adopt a smooth-funneled energy landscape, avoiding energetic conflicts that exhibit frustration.^57–60^ However, local frustration is present in the protein to perform essential functions.^61–66^ In this work, we explored the relationship between non-native interactions, the presence of stable intermediates, and the residual frustration observed in the NTD.

We employed the ‘Frustratometer’ web server to compute local frustration in the native structure and the intermediates. According to the principle of minimal frustration, contacts in the native state should be minimally frustrated, *i.e.* energetically favorable. It has been previously shown that most of the native contacts in globular proteins are minimally frustrated (∼40%), while 10% contacts are highly frustrated in the native state.^63,67^ These highly frustrated contacts map to functional sites and are thought to be evolutionarily conserved.^68^

**Fig. 8:**
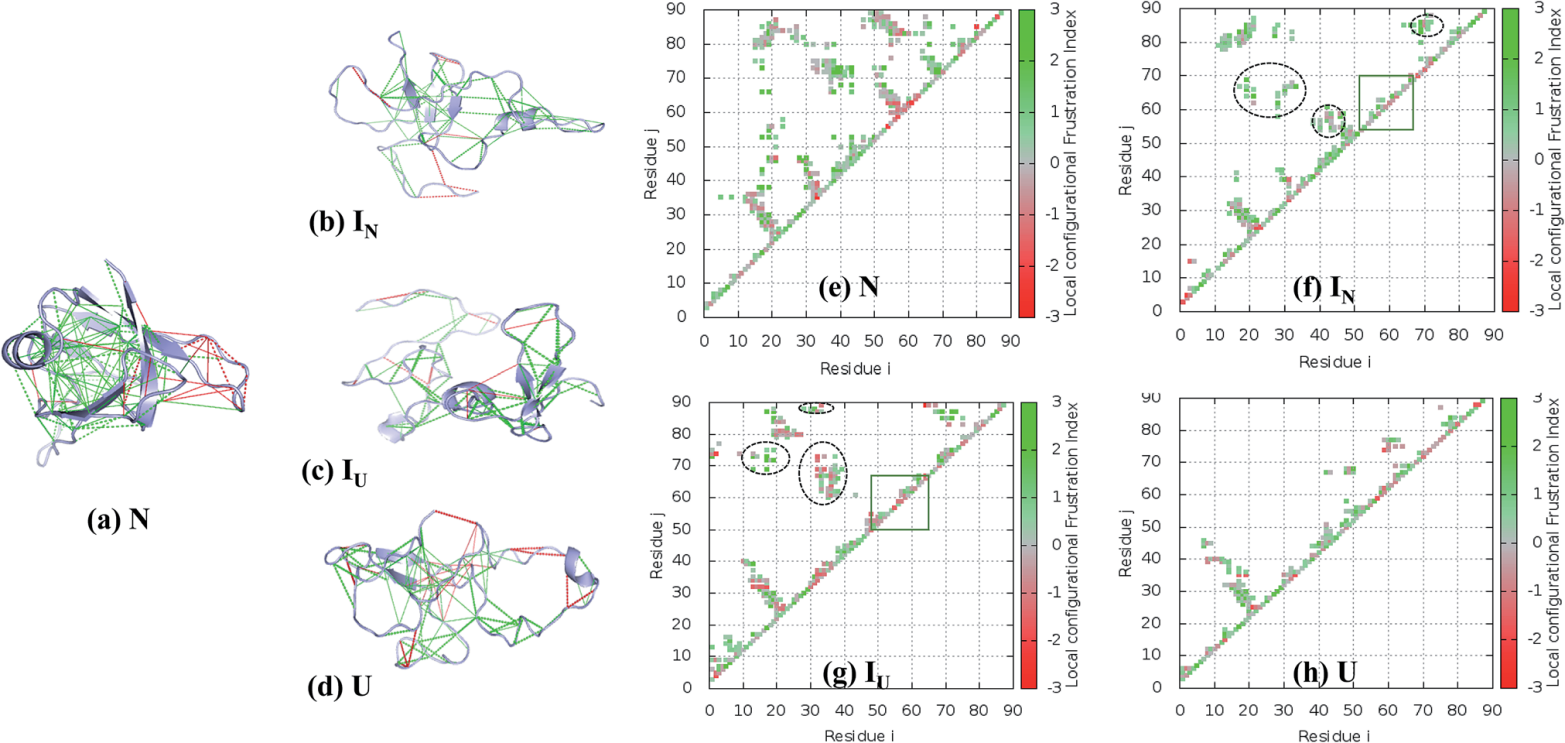
Residual frustration analysis in the selected states of the NTD. (a) Local frustration is depicted on the native NTD structure (PDB : 2N4P). The large cluster of minimally frustrated interactions (green) defines the core of the protein, whereas some highly frustrated interactions (red) occur on the surface of the protein. Local frustration in the (b) I_N_, (c) I_U_, and (d) U states depicting the large changes in frustration profiles. (f–h) Contact maps of the corresponding transitions of the NTD compared with the native state, along with their frustration profiles. The distinct non-native clusters in the intermediate states are circled in black. Within the β-hairpin region (residues 55–62), many frustrated contacts remain unformed in the intermediate states (orange box). (e) Energetically favorable residue pair contacts of NTD.

We calculated the configurational frustration index of the NTD, as shown in Fig. 8a. It can be seen that the NTD is less minimally frustrated compared to typical proteins. The results show that among all the native contacts, 26% are minimally frustrated (Fig. 8a, green line), compared with 37% in a typical protein, as shown by Ferreiro *et al.*^63^ However, the minimally frustrated contacts form a cluster around the protein core, as is observed in most proteins. The protein core consists of four β-sheets (β1 to β4) and an α-helix. Importantly, the highly frustrated contacts comprise 5% of the total contacts, which is less than what is expected in a globular protein (10%) (Fig. 8a, red line). A single large highly frustrated contact cluster is found in the β-hairpin region (residues 55–65) between β3 and β4. This region is unique in the NTD, and the nearby β5 strand is linked to the rest of the protein through H-bonds and hydrophobic interactions. Taken together, the NTD has fewer frustrated contacts and fewer minimally frustrated contacts, suggesting a different energy landscape compared to typical proteins.

The FEL of the NTD in 8 M urea at the transition temperature (350 K) indicates the presence of highly populated long-lived intermediates, I_N_ and I_U_, during the 500 ns simulation run (Fig. 5a and b). The intermediate, I_N_, with *R*_g_ ∼ 1.3 nm is unusually compact, and the structure generally contains a three-stranded β-sheet core (β1, β2, and β6) (Fig. 8b). Meanwhile, the second intermediate ensemble, I_U_, contains a two-stranded β-sheet core (β1 and β2) (Fig. 8c). The I_U_ ensemble displays a higher number of highly frustrated contacts (10%, red line) compared to I_N_ (5%) and fewer minimally frustrated contacts (24%, green line) than I_N_ (28%). The unfolded ensemble, U, displays more highly frustrated contacts (Fig. 8d).

To establish how the contact maps of the unfolding intermediates display residual frustration, we plotted the contact maps according to their frustration indices; these are shown in Fig. 8e–h. In the first intermediate structure, I_N_, residues of the α-helix (40–43) form many non-native contacts with β2 (28–29), β3 (55) and β-hairpin residues (57–59) (Fig. 8f, circled black dots) compared to the N structure (Fig. 8e). In addition, Val69 of β4 interacts non-natively with the residues of the loop region between β5 and β6 (80–84), Tyr 85 and Val 86 of β6. The residual local frustration reveals that these non-native contacts are minimally frustrated (Fig. 8b) and can stabilize the intermediate state through energetically favorable contacts that are not observed in the native state.

Similarly, the I_U_ ensemble also displays the formation of non-native contacts between β1 (17–19) and Val69 (β4), Ile72 (β5) and Val87 (β6) (Fig. 8g, circled black dots). Also, β6 (87–88) interacts non-natively with the loop region (32–35) between β2 and the α-helix. These non-native contacts are minimally frustrated, imparting stability to the I_U_ state. In addition to favorable interactions, I_U_ also exhibits highly frustrated non-native contacts involving the loop region (32–39) and β-hairpin (55–65) (Fig. 8g).

Thus, whereas the core of the structure is stabilized by both native and non-native favorable interactions, the β-hairpin structure surrounding β3 and β4 does not show any particular native or non-native contacts in either I_N_ or I_U_ (Fig. 8f–g, orange box). Moreover, in this region, favorable interactions are absent and some highly frustrated interactions are found. We therefore expect that both the presence of local frustration and the rugged landscape of this β-hairpin region play important roles in the stabilization of the unfolding intermediates of the NTD. Indeed, this region behaves very similarly in the unfolding ensemble, as can be seen in Fig. 8h.

## Conclusion

Structural integrity of the NTD is required for the proper function of TDP-43. The unfolded state of the NTD together with the disordered prion-like domain initiates irreversible aggregation of TDP-43, causing disease. A detailed account of the unfolding and thermodynamics behaviors of the NTD has been delineated using MD simulations. The cumulative data of ∼2.75 μs simulations enabled us to sample the complex conformational energy landscape of the NTD, which is characterized by the presence of multiple intermediates along the unfolded conformation at 350 K and the population of completely unfolded species at higher temperatures (400 K to 500 K). We found a rugged free energy basin surrounded by different metastable conformational states with comparable stabilities and a low energy barrier in 8 M urea at 350 K. At this temperature, we observed that during the early stage of unfolding, the initially solvent-buried W80 (and Y55) become solvent-exposed, giving rise to a native-like molten globule state, I_N_. I_N_ eventually unfolds to I_U_, which is characterized by transitions between partial burial and exposure of W80 during unfolding, indicating conformational heterogeneity in I_U_. The structural plasticity of the environment around W80 in the I_U_ ensemble results in the formation of non-native, tertiary interactions with exposure of the hydrophobic core. The secondary structure evolution also reveals the high stability of N-terminal β1 and β2 in I_U_ and even the U ensemble. Moreover, local frustration and favorable non-native interactions stabilize the I_U_ state. Thus, the simulation results suggest that non-native interactions along with persistence of the residual structure and hydrogen bonds during the unfolding of the NTD domain may mediate irreversible aggregation of the protein.

## Conflicts of interest

The authors declare no conflict of interest.

## Supplementary Material

RA-008-C8RA03368D-s001
