# Supplementary material for: Elucidation of the structural stability and dynamics of heterogeneous intermediate ensembles in unfolding pathway of the N-terminal domain of TDP-43
Source: RSC Adv. 2018 May 30;8(35):19835–45. doi: 10.1039/c8ra03368d (PMC9088055; doi:10.1039/c8ra03368d)
Supplement: RA-008-C8RA03368D-s001 [file RA-008-C8RA03368D-s001.pdf]

Electronic Supplementary Material (ESI) for RSC Advances.
This journal is © The Royal Society of Chemistry 2018

Supporting Information

Elucidation of the Structural Stability and Dynamics of Heterogeneous Intermediate Ensembles in Urea Induced Unfolding of the N-Terminal Domain of TDP-43

Amresh Prakash[1]*, Vijay Kumar[2] *, Naveen Kumar Meena[1] and Andrew. M. Lynn[1]

[1]School of Computational & Integrative Sciences, Jawaharlal Nehru University, New Delhi-110067, India

[2]Centre for Interdisciplinary Research in Basic Sciences, Jamia Millia Islamia, New Delhi-110025, India

Table S1.

Run id	Temp. (K)	length (ns)	RMSD (highest) (nm)	U visited	MG visited	IU visited
01	300*	500	1.24	No	No	No
02	300	500	1.44	No	No	No
03	350	500	2.16	Yes	Yes	Yes
04	400	500	2.78	Yes	No	No
05	450	500	2.84	Yes	No	No
06	500	250	3.65	Yes	No	No

All simulations were performed in aqueous 8 M urea solution at 1 atm pressure. The population of IU was determined on the basis of the following criterion: $0.25 \leq Q \leq 0.5$. *The simulation carried out in pure water at 300 K for the period of 500 ns.

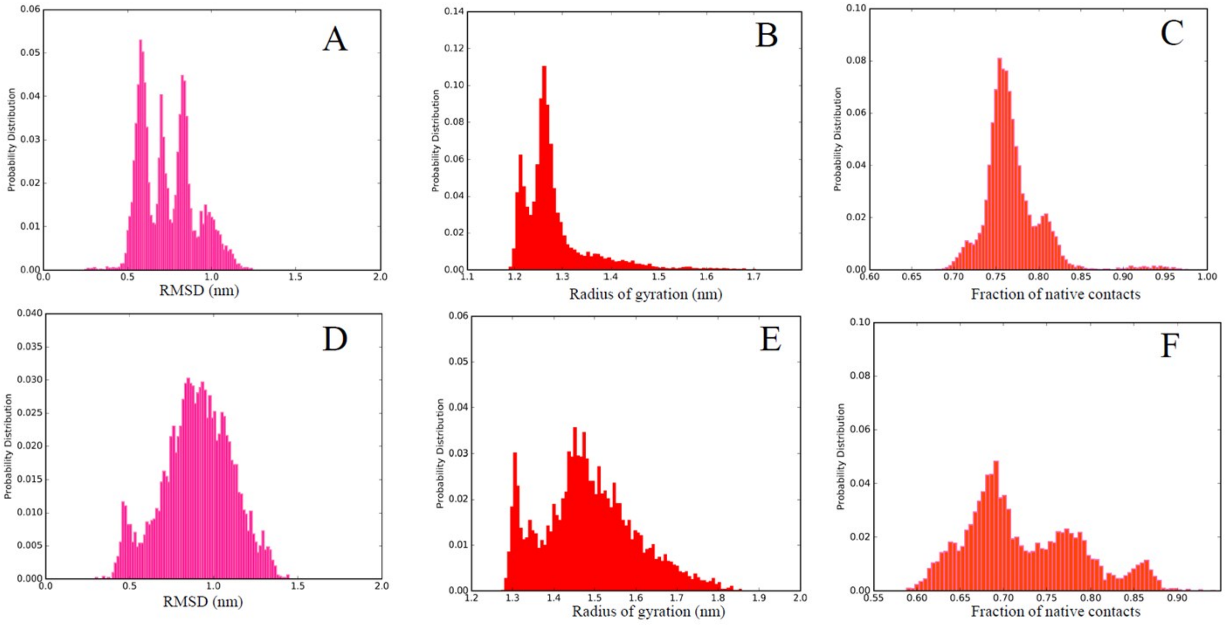

Figure S1. Probability distribution of RMSD, Rg, Nc of NTD in water (A-C) and in 8M urea (D-F) at 300 K.

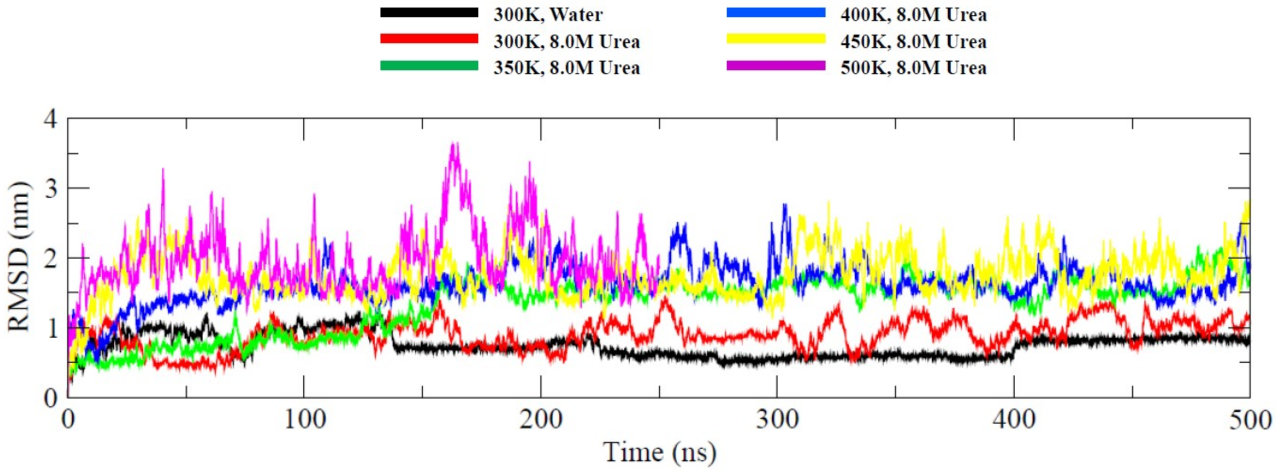

Figure S2A. Time evolution plot of all-atoms C_α RMSD in water and 8 M urea at different temperatures.

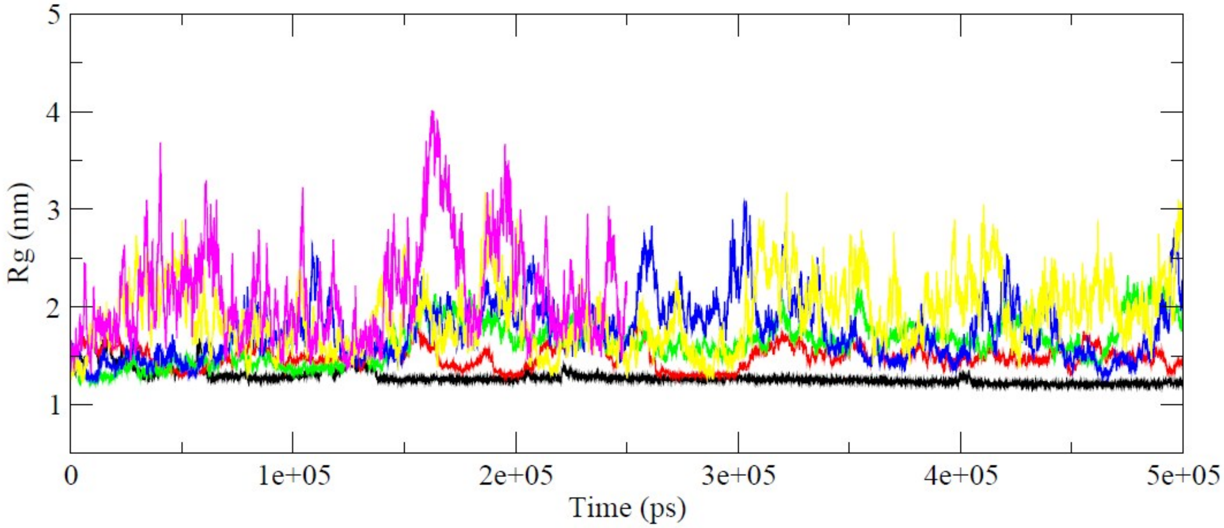

Figure S2B. Time evolution plot of radius of gyration (R_g) of all C_α atoms in water and 8 M urea at different temperatures. Color scheme as labelled above.

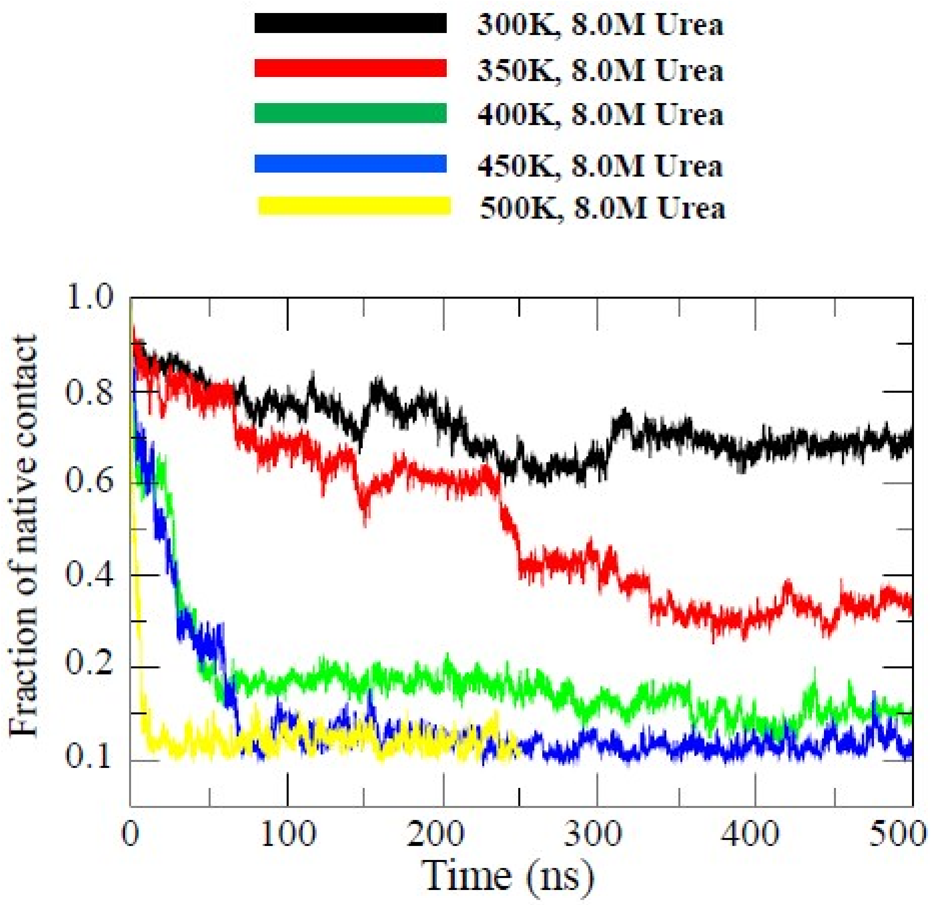

Figure S2C. Time evolution of the fraction of native contact in 8 M urea at different temperatures (300-500K). Color scheme as labelled above.

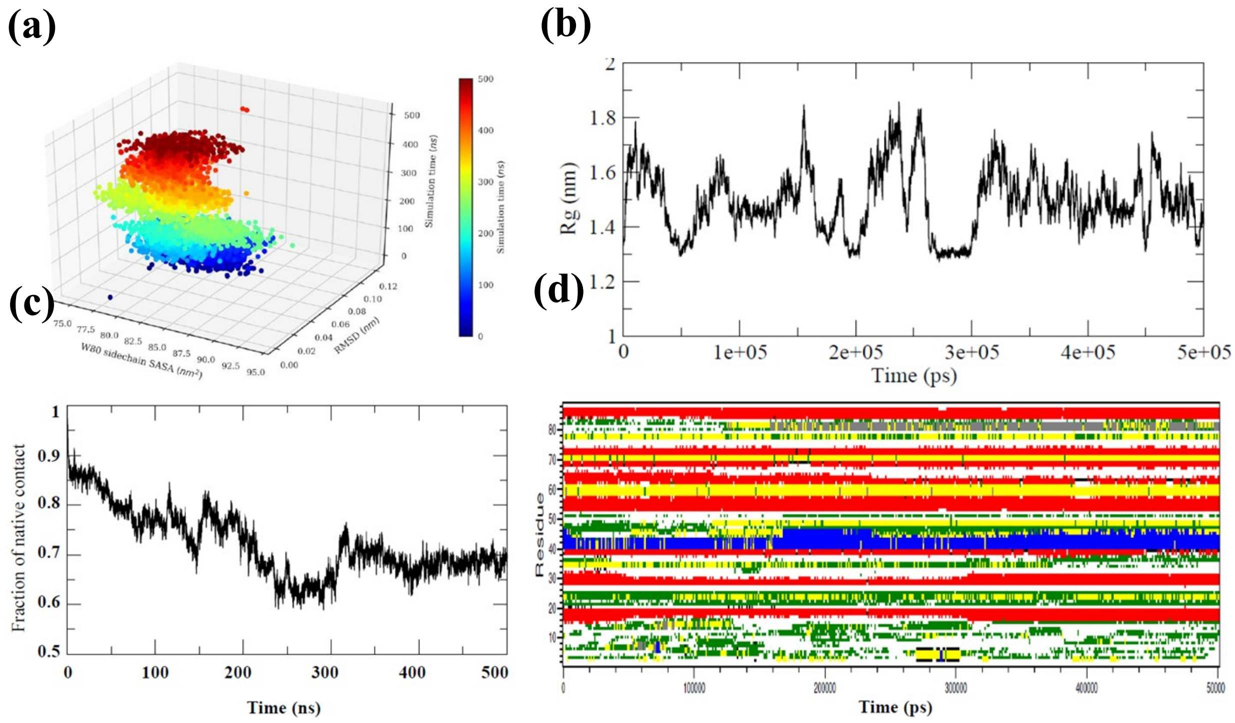

Figure S3. Unfolding pathways of NTD in 8 M urea at 300 K. (a) Evolution of the solvent-exposed surface area, SASA of W80 side chain and Cα- RMSD during unfolding. Each point on this plot is colored according to its time of occurrence, with the color scale shown. Time evolution of (b) R_g, (c) Nc, and (d) secondary structure.

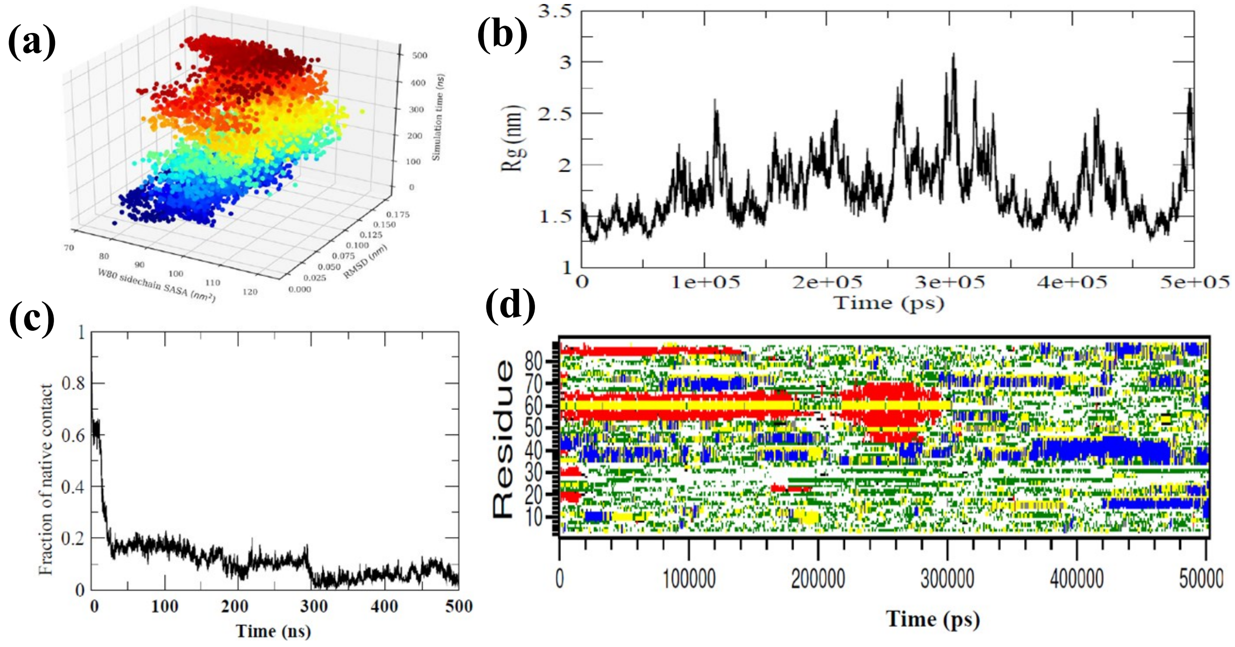

Figure S4. Unfolding pathways of NTD in 8 M urea at 400 K. (a) Evolution of the solvent-exposed surface area, SASA of W80 side chain and Cα- RMSD during unfolding. Each point on this plot is colored according to its time of occurrence, with the color scale shown. Time evolution of (b) R_g, (c) Nc, and (d) secondary structure.

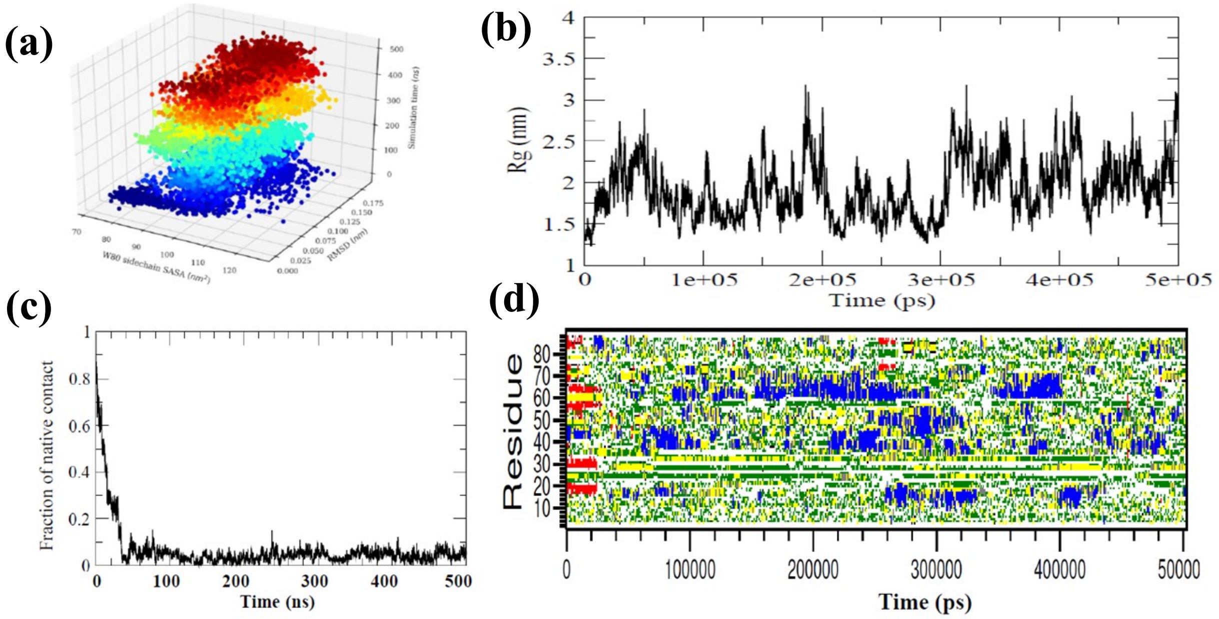

Figure S5. Unfolding pathways of NTD in 8 M urea at 450 K. (a) Evolution of the solvent-exposed surface area, SASA of W80 side chain and Cα- RMSD during unfolding. Each point on this plot is colored according to its time of occurrence, with the color scale shown. Time evolution of (b) R_g, (c) Nc, and (d) secondary structure.

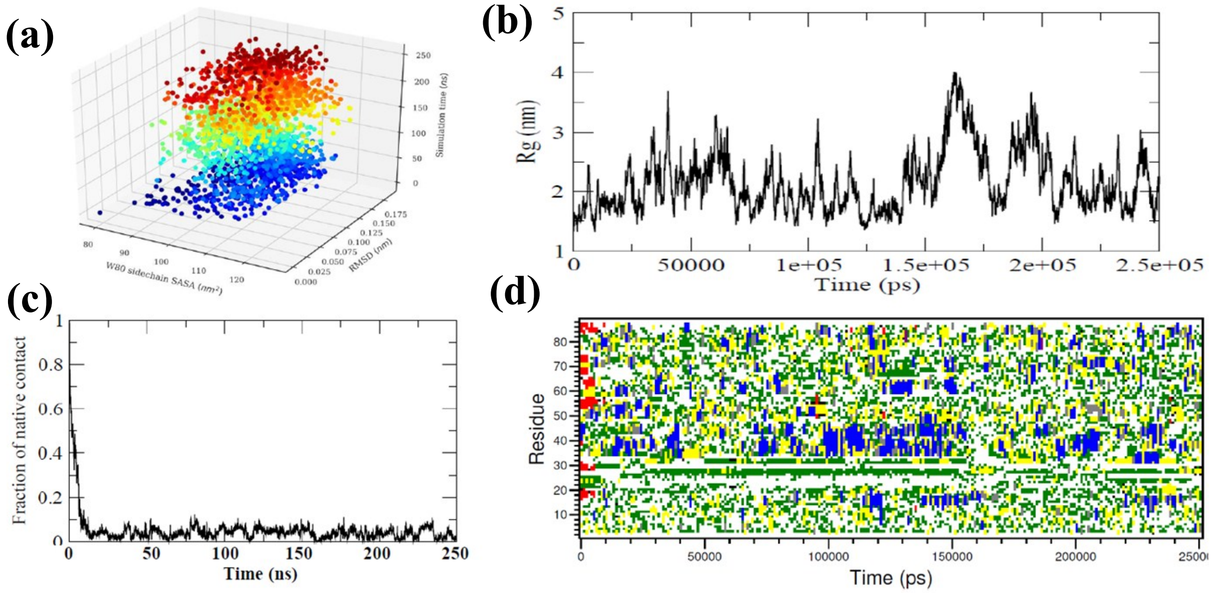

Figure S6. Unfolding pathways of NTD in 8 M urea at 500 K. (a) Evolution of the solvent-exposed surface area, SASA of W80 side chain and Cα- RMSD during unfolding. Each point on this plot is colored according to its time of occurrence, with the color scale shown. Time evolution of (b) R_g, (c) Nc, and (d) secondary structure.

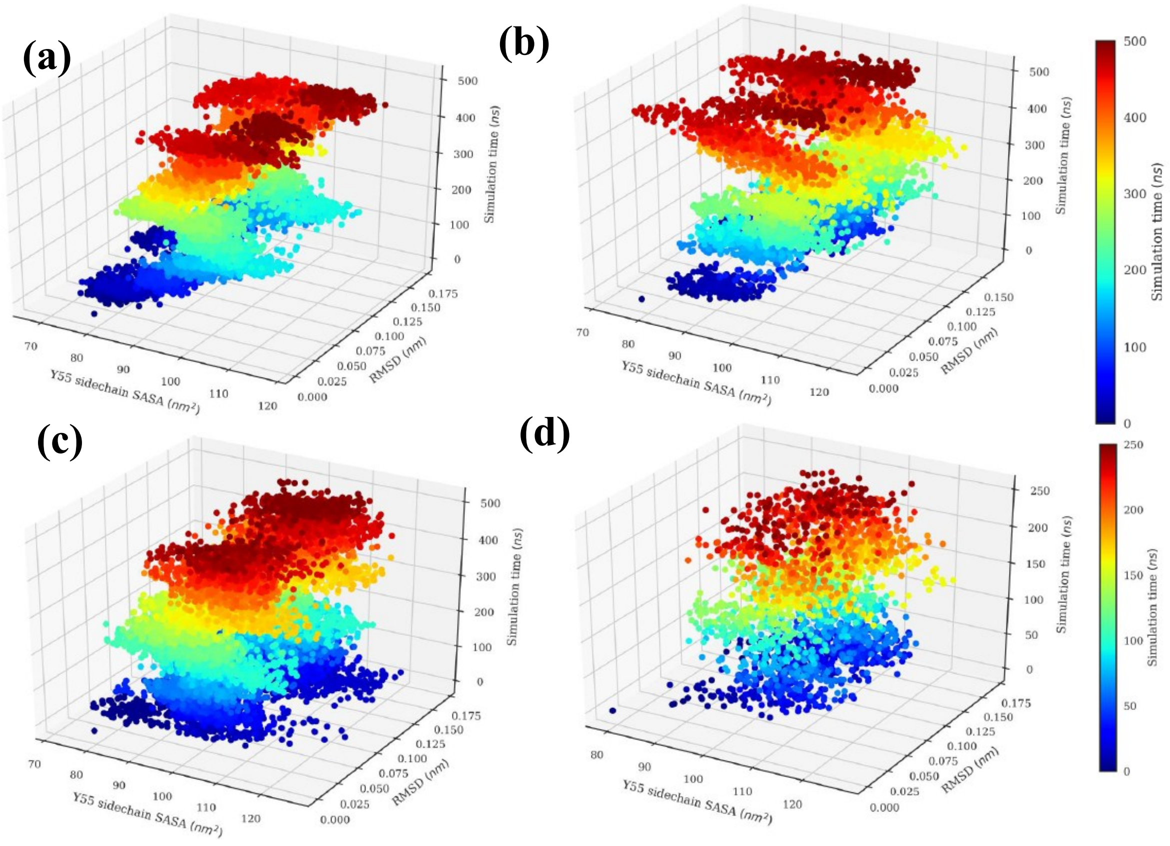

Figure S7. Time evolution of C_α RMSD and SASA of side chain of Y55 in 8M urea at (a) 350 K, (b) 4000 K, (c) 450 K and (d) 500 K. Each point on this plot is colored according to its time of occurrence with the color scale shown.

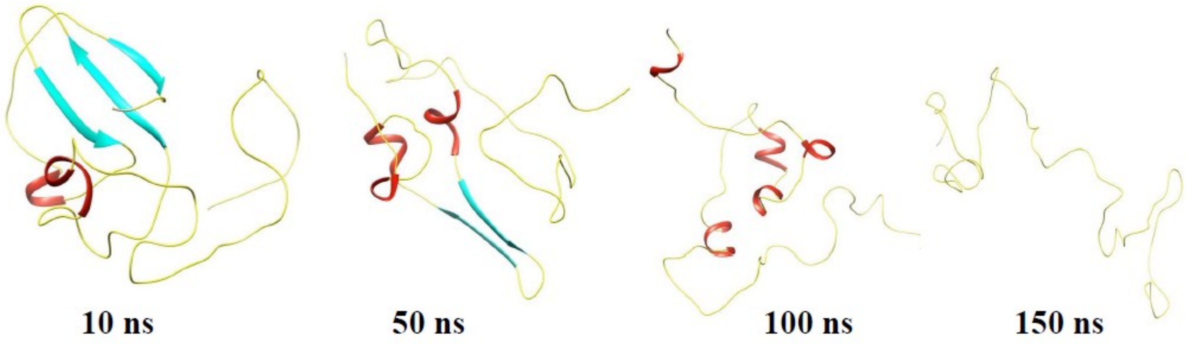

Figure S8. Snapshots of representative conformational states observed during unfolding pathway of NTD in 8 M urea at 400K.

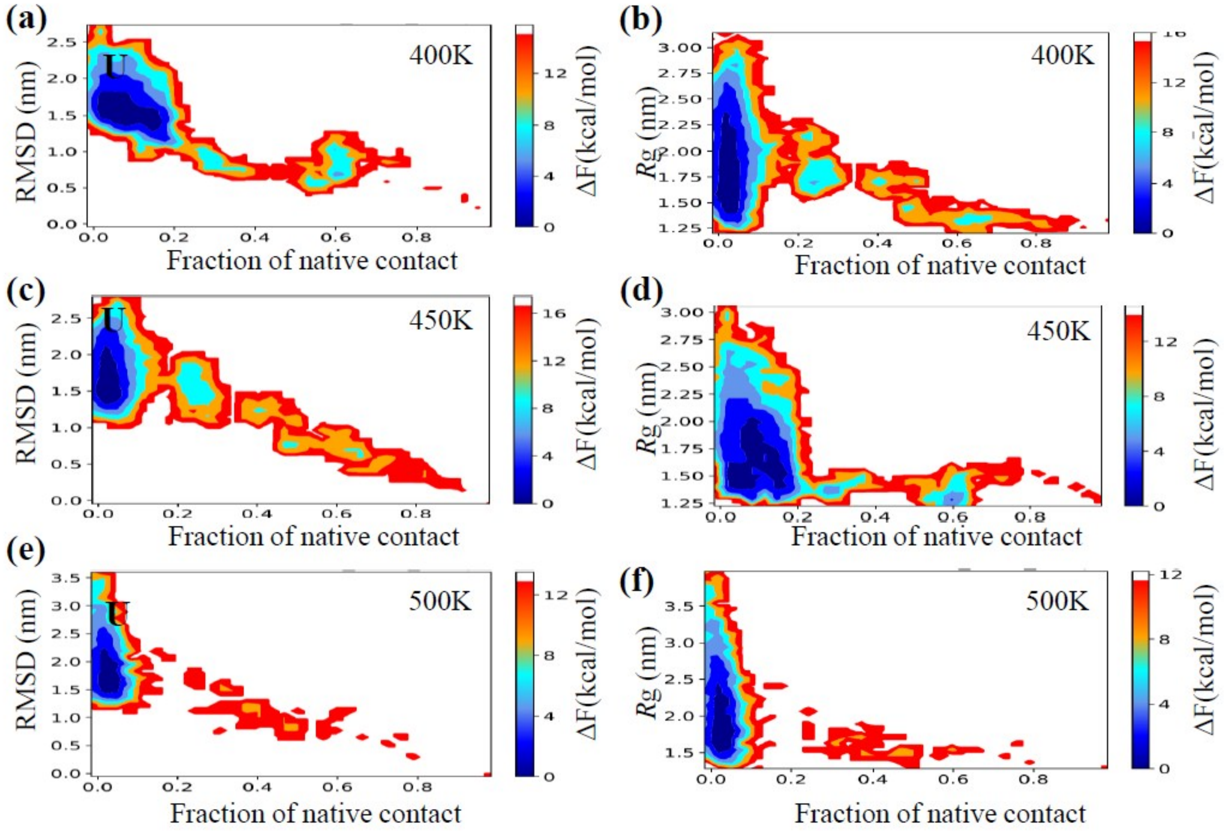

Figure S9. Free-energy landscape of unfolding dynamics of NTD at higher temperature. At higher temperatures (400K-500K) protein unfolded readily, as plot shows the single energy basin populated with unfolded conformations having almost complete loss of native contacts.

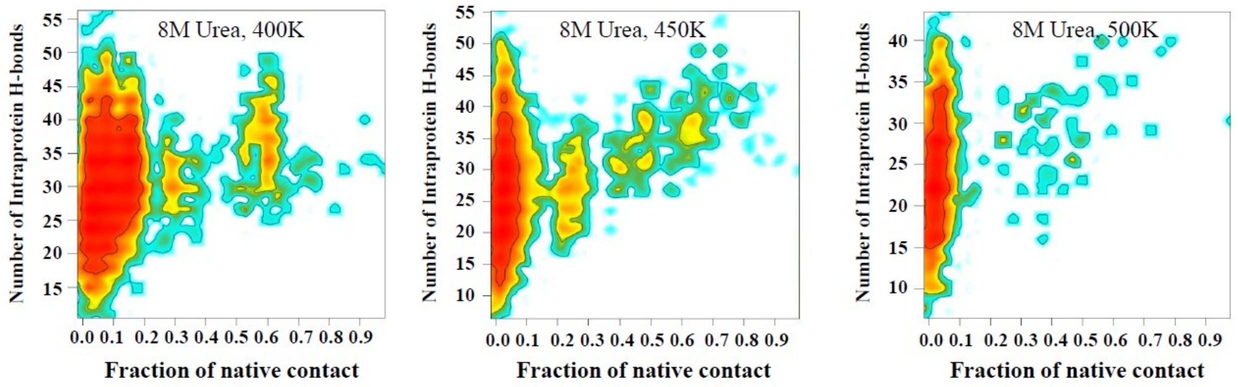

Figure S10. Free-energy landscape involving the fraction of native contacts vs number of intraprotein H-bonds in denaturant (8M Urea) at higher temperatures (400K, 450K and 500K). Plots showed readily loss of h-bonds that is provided the clear evidence of rapid unfolding at higher temperatures.

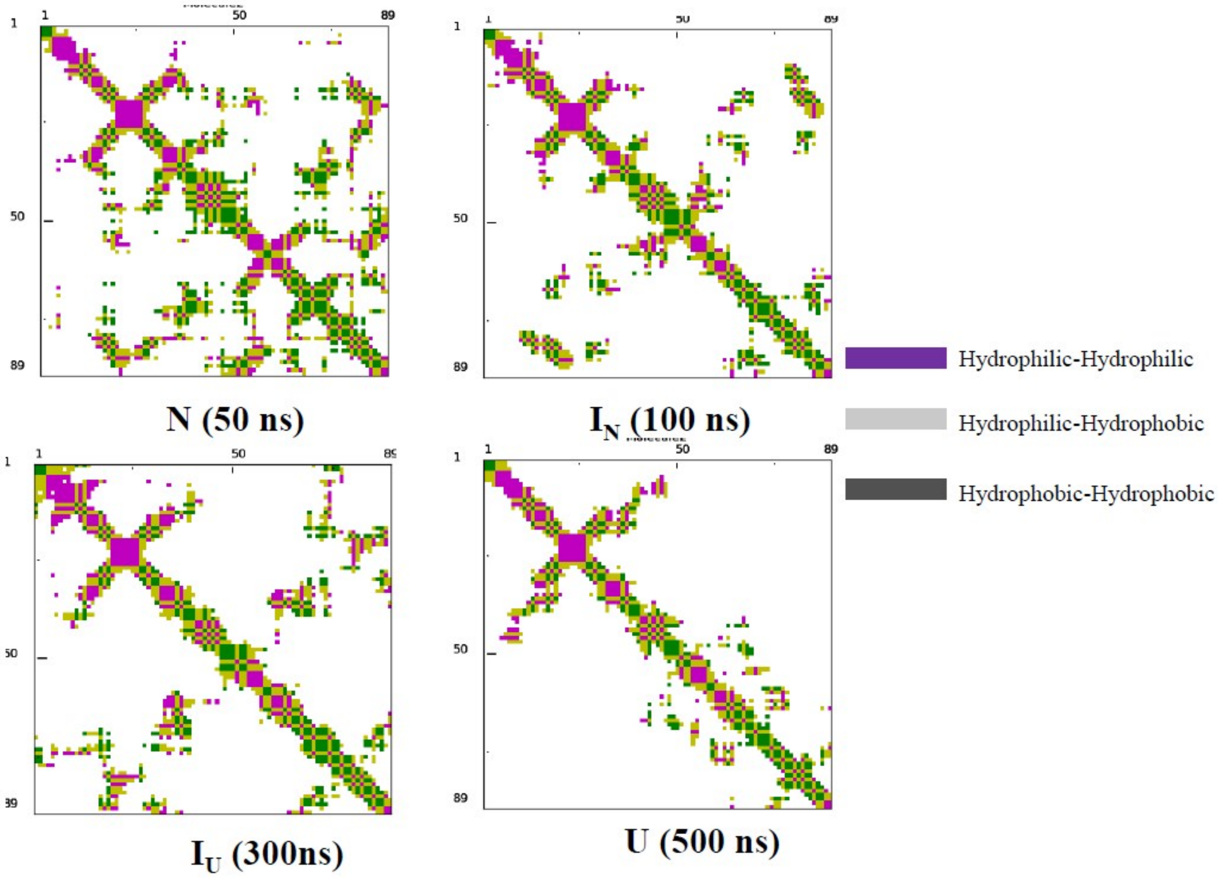

Figure S11. Inter-residual contact map of the different unfolded states of NTD obtained from the MD simulation plotted by COCOMAPS.
